# Flatworm mucus as the base of a food web

**DOI:** 10.1186/s12898-019-0231-2

**Published:** 2019-03-29

**Authors:** Benjamin Wilden, Nabil Majdi, Ute Kuhlicke, Thomas R. Neu, Walter Traunspurger

**Affiliations:** 1 Department of Animal Ecology, University of Bielefeld, Konsequenz 45, 33615 Bielefeld, Germany; 2 EcoLab, UMR 5245, CNRS, INP, UPS, ENSAT, Université de Toulouse, 118 route de Narbonne, 31062 Toulouse, France; 3 Department River Ecology, Helmholtz Centre of Environmental Research, Brückstr. 3a, 39114 Magdeburg, Germany

**Keywords:** Ecological engineering, Mucus structure, Confocal microscopy, Aquatic ecology, Niche construction

## Abstract

**Background:**

By altering their habitats, engineering species can improve their own fitness. However, the effect of this strategy on the fitness of coexisting species or on the structure of the respective food web is poorly understood. In this study, bacteria and bacterivorous nematodes with short (*Caenorhabditis elegans*) and long (*Plectus acuminatus*) life cycles were exposed to the mucus secreted by the freshwater flatworm *Polycelis tenuis.* The growth, reproduction, and feeding preferences of the nematodes in the presence/absence of the mucus were then determined. In addition, confocal laser scanning microscopy (CLSM) was used to examine the structural footprint of the mucus and the mucus colonization dynamics of bacteria and protozoans.

**Results:**

Mucus exposure resulted in a greater reproductive output in *P. acuminatus* than in *C. elegans*. In a cafeteria experiment, both nematode species were attracted by bacteria-rich patches and were not deterred by mucus. CLSM showed that the flatworms spread a layer of polysaccharide-rich mucus ca. 15 µm thick from their tails. Subsequent colonization of the mucus by bacteria and protozoans resulted in an architecture that progressively resembled a complex biofilm. The presence of protozoans reduced nematode reproduction, presumably due to competition for their bacterial food supply.

**Conclusion:**

Animal secretions such as mucus may have broader, community-level consequences and contribute to fueling microbial food webs.

**Electronic supplementary material:**

The online version of this article (10.1186/s12898-019-0231-2) contains supplementary material, which is available to authorized users.

## Background

By constructing physical structures or modifying pre-existent habitats, organisms can actively modify biogeochemical gradients. If the effect is beneficial and maintained over time and throughout population turnover, the ecological success and evolutionary prospects of the engineering species will be favored. The process that defines this ecological engineering behavior is referred to as “niche construction” and it can be observed on scales ranging from the extremely local to the global [1, 2]. Niche construction links ecological inheritance with evolutionary concepts. In other words, organisms transmit not only their genes to their offspring, but also the environment they built/modified during their life. Typical examples are vertebrates that build elaborate nests or burrows; social insects that practice nest maintenance, defense and regulatory behaviors [3, 4]; and humans, who are perhaps the most notorious niche constructors [5].

However, niche construction effects can extend far beyond those that are beneficial for the niche constructor. By increasing environmental complexity, niche-constructing organisms may impose ecologically and evolutionary relevant constraints on other species. There is mounting evidence that niche construction strongly impacts sympatric species, by creating an additional selective pressure that can shape macro-evolutionary patterns over geological time [6, 7]. However, the extent to which niche construction induces positive and negative feedbacks at the level of complex communities and food webs is often difficult to assess [8].

In this study we conducted a set of laboratory-controlled experiments using a freshwater flatworm species (*Polycelis tenuis*, Ijima 1884) that secretes a viscid mucus for locomotion and to trap its prey (e.g., nematodes) [9, 10]. Previous studies demonstrated the engineering effects of freshwater flatworm mucus [11, 12]. For example, in a field enclosure study [12], we showed that a freshwater flatworm (*Polycelis felina*) maintained the local availability of its prey (midge larvae) by increasing the availability of both prey habitat (fine sediments) and prey resource (nematodes and bacteria) on the leaf litter surface. We found that upward stimulation of the food web was primarily driven by a higher biomass of bacteria and nematodes in the leaf packs that included flatworms. By contrast, in a similarly designed experiment using similarly sized predators (stonefly larvae) that did not secrete mucus, there was no upward stimulation of the detritus-based food web [13]. “Mucus-gardening” may increase flatworm fitness by reducing both the prey searching time and intra-specific competition. It can thus be regarded as evidence of a niche construction effect that transmits upwards throughout detritus-based food webs and may impact numerous coexisting species. Furthermore, in a laboratory experiment performed in sediment microcosms, the presence of flatworms was found to affect phosphorus availability, by increasing the biomass of heterotrophic bacteria, flagellates, and ciliates [14]. These results suggest that flatworm mucus has both physical and chemical effects.

The aim of the present study was to test the effects of flatworm mucus on other species and, more broadly, on the food web. We therefore monitored the consequences of mucus deposition for a community composed of nematodes and microbes coexisting (or not) in the field with the flatworm *P. tenuis*. We expected that nematode fitness would be affected, either negatively or positively, by the flatworms’ mucus, e.g., through a bottom-up stimulation of bacterial abundance. Nematodes are able to sense various chemical cues, even perhaps the presence of a predator [15, 16]. Similarly, if mucus has an impact on nematode fitness, nematodes should be able to sense its presence and then either avoid or exploit it. We also investigated the extent to which mucus trails represent a durable structure and resource for other prokaryotic and eukaryotic organisms.

## Results

### Nematode bioassay

**Fig. 1:**
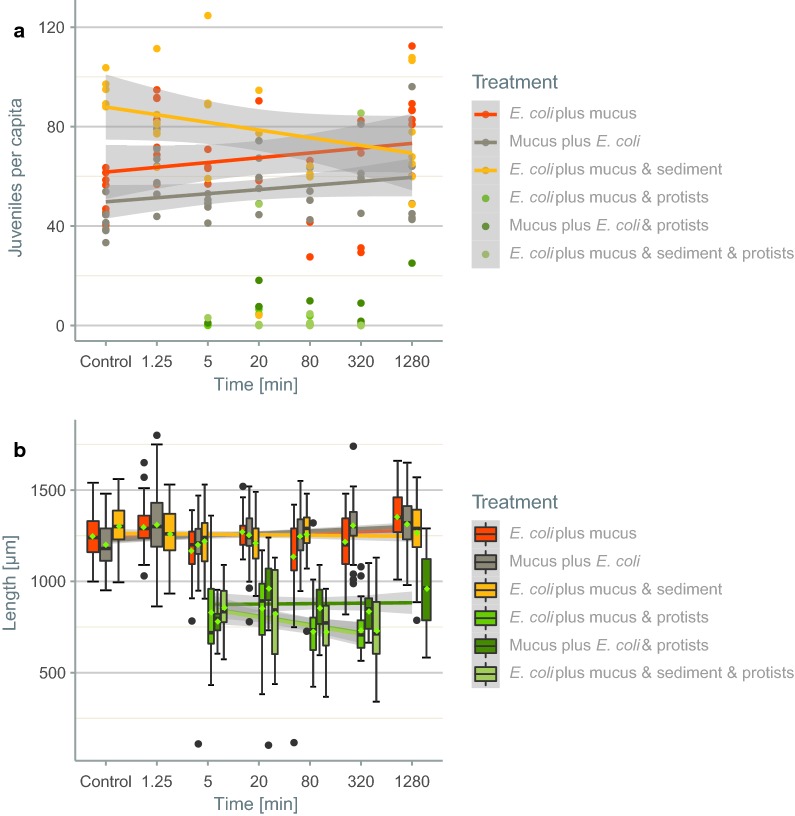
Juveniles per capita (**a**) and body length [µm] (**b**) in the nematode bioassay. *Caenorhabditis elegans* was incubated for different exposure times with *Polycelis tenuis* in the presence or absence of *Escherichia coli,* as well as in a sediment setup (**a** N = 7 per time point and treatment; **b** N = 1629). Green points in **a** indicate protozoans. The data are presented in box-whisker plots (**b**); the median is indicated by the horizontal line, and the mean by the green rhombus (**b**). The boxes show the interquartile range, and the whiskers either the 5% or 95% percentile. In addition to the actual data, the fit obtained with linear mixed effect models (LMMs) is shown together with the 95% confidence interval (**a** R^2^c = 0.06; **b** R^2^c = 0.42)

The effect of flatworm mucus on nematode fitness was investigated in a bioassay using the nematode *C. elegans*. The results showed that the number of juveniles per capita did not differ across treatments or with respect to mucus deposition time, nor was the interaction of the two factors significant (Additional file 1: Table S1). All protozoans in the vials were identified as *Tetrahymena pyriformis* (Additional file 1: Fig. S2). In the presence of protozoans, the number of *C.* *elegans* offspring declined by > 75% (Fig. 1a), in significant contrast to the reproductive output of the nematode in the absence of protozoans (*t*-test, df = 141, t = − 15.944, P < 0.001). The body length of *C. elegans* did not differ significantly between treatments but it was reduced by about 35% in the presence of protozoans (Fig. 1b; Additional file 1: Table S2).

**Fig. 2:**
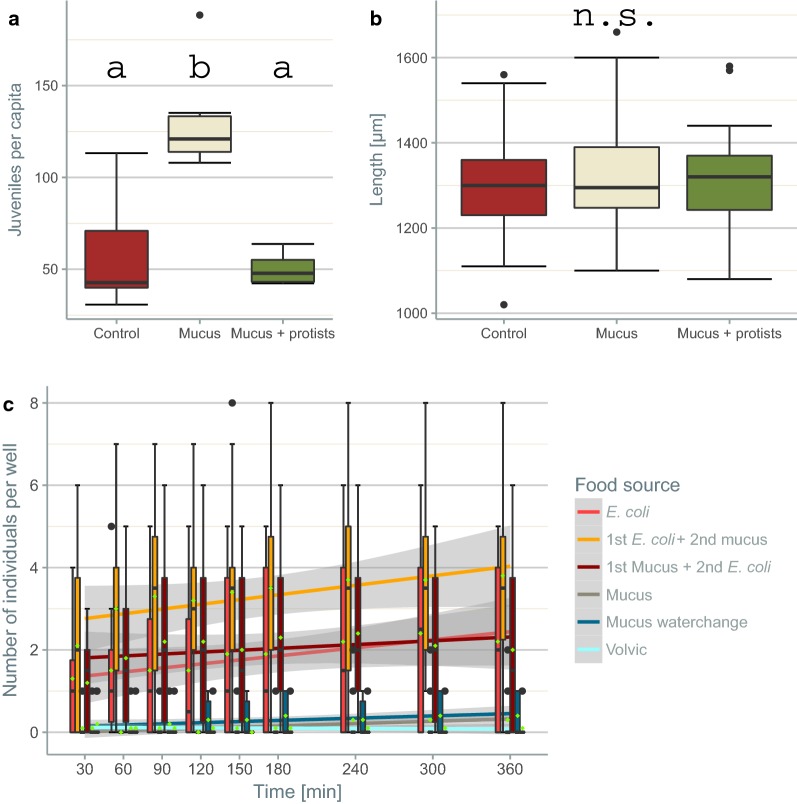
Juveniles per capita (**a**) and body length [µm] (**b**) in the nematode bioassay as well as individuals per food source in the cafeteria experiment (**c**). *Plectus acuminatus* was incubated with and without the mucus of *P. tenuis* as well as protozoans (**a**, **b**). Number of *C.* *elegans* (**c**) per well containing *E. coli*, *E. coli* followed by mucus, mucus followed by *E. coli,* mucus, mucus with a water change or water. The data are presented in box-whisker plots. The median is indicated by the horizontal line, and the mean by the green rhombus (**c**). The boxes show the interquartile range, and the whiskers either the 5% or 95% percentile. In addition to the actual data, the fit obtained with LMMs is shown together with the 95% confidence interval (**c** R^2^c = 0.41). The results of a post hoc pairwise Wilcoxon rank sum test between the treatments are indicated as different letters for differences determined to be significant (**a** N = 20; **b** N = 200)

In the nematode bioassay with *P.* *acuminatus*, the number of juveniles per capita more than doubled when mucus was available (Fig. 2a; Kruskal–Wallis test, X^2^ = 11.547, P < 0.01). In treatments containing both mucus and protozoans, however, the increase in reproductive output was not significant (post hoc pairwise Mann–Whitney-U test; after Bonferroni-Holm correction: P = 0.671). Body length did not differ across treatments (Fig. 2b; Kruskal–Wallis test, X^2^ = 1.3752, P = 0.50).

### Cafeteria experiment

A cafeteria experiment was performed to determine whether nematodes sense the presence of mucus and then either avoid or exploit it. Both nematode species were offered different food sources to choose from: Volvic, mucus in Volvic, mucus in Volvic and replacement of the water after flatworm contact, mucus in Volvic and replacement of the water with an *E. coli* suspension after flatworm contact, mucus in *E. coli* suspension and pure *E. coli* suspension. The results showed a clear preference of *C.* *elegans* for the bacteria-containing wells, rather than for non-bacteria-containing wells (Fig. 2c; Additional file 1: Table S6). Moreover, wells in which the flatworms had direct contact with *E. coli* were even more attractive to the nematode.

In the case of *P.* *acuminatus,* the number of nematodes per food source offered, increased very slowly and did not reach a plateau during the experiment. Nonetheless, a treatment effect (Additional file 1: Table S4) was evidenced by the significant difference in the number of nematodes exposed to mucus + *E. coli* vs. mucus as a stand-alone food source (Additional file 1: Table S7).

### Mucus composition and structure

The results of the study of the lectins are as follows: (i) no staining (HPA, PSA, VVA), (ii) indirect staining due to associated particles and cells (WGA), (iii) weak staining (Ban, GS I) and stronger staining (AAL, RCA). Since the signal of the RCA lectin was the strongest, RCA was employed as stain in most experiments. According to the supplier’s data sheet, RCA has a specificity for galactose or *N*-acetylgalactosamine residues.

**Fig. 3:**
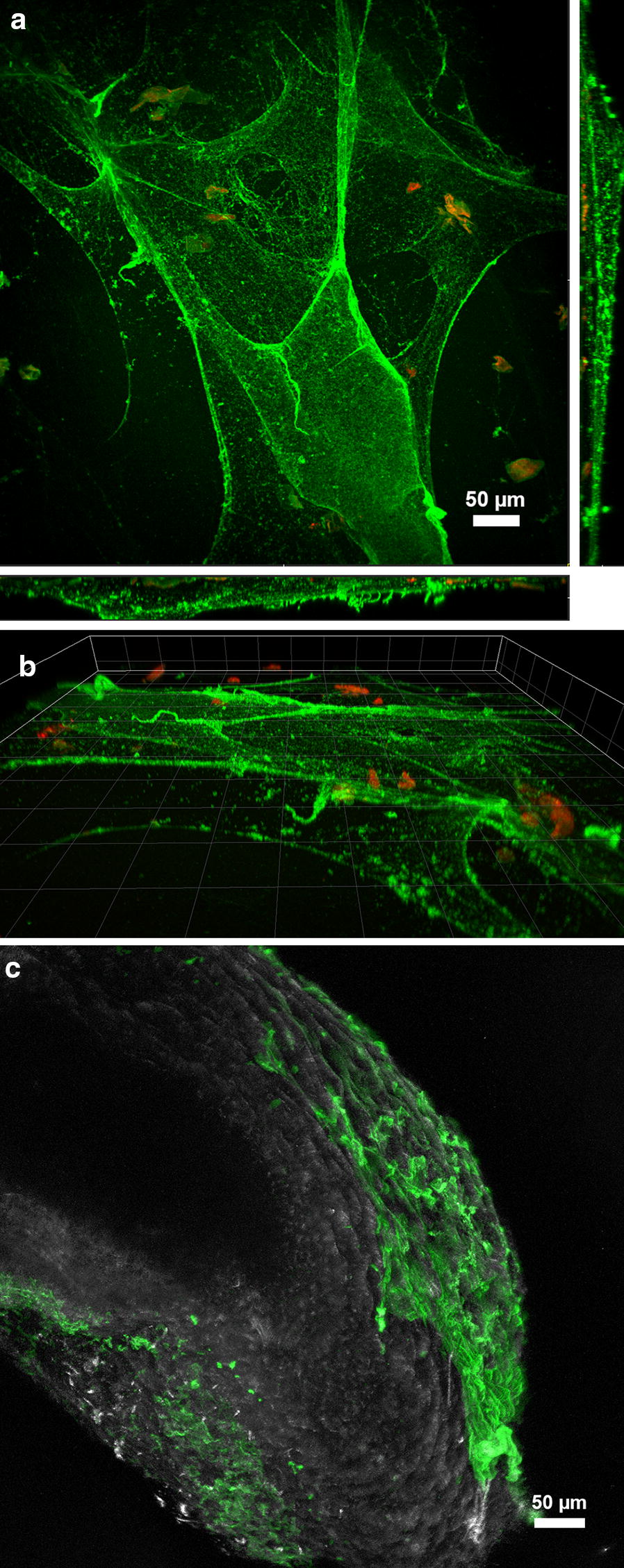
Confocal microscopy of mucus after staining with the glycoconjugate specific lectin RCA-FITC (**a**–**c**) and the nucleic acid-specific fluorochrome Syto60 (**a**, **b** only). The images in **a** and **b** are derived from the same dataset. **a** XYZ maximum intensity projection illustrating the top and side views of the lectin-stained mucus sheet. **b** 3D transparent projection indicating the spatial distribution of the thin individual mucus sheets. **c** Confocal image showing the tail of a flatworm with the lectin-stained mucus attached to the animal surface. Color allocation: in **a**, **b**, **c** lectin = green, in **a**, **b** nucleic acids = red (seems to stain detrimental objects), in **c** reflection = grey

**Fig. 4:**
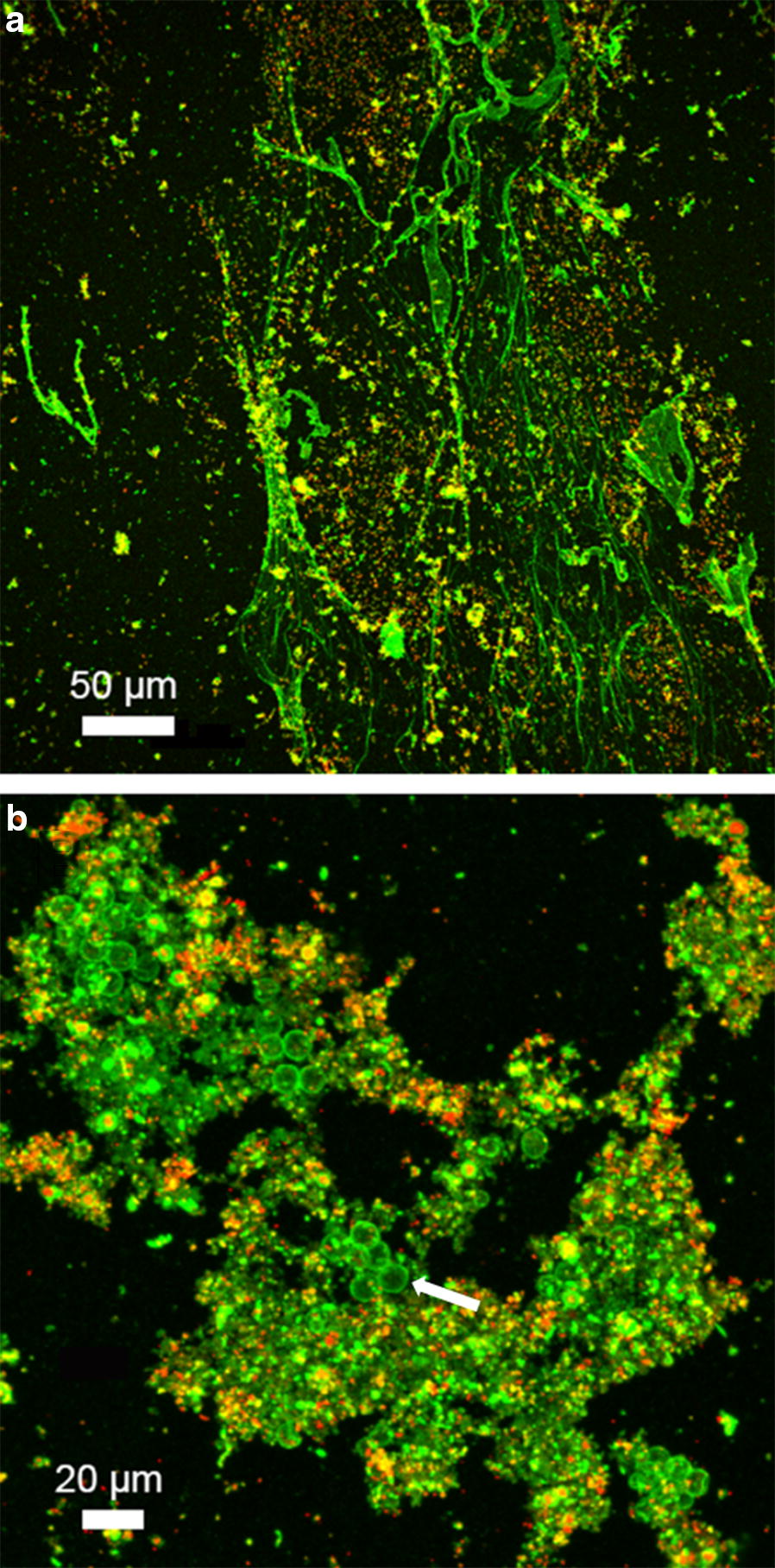
Confocal data sets of colonized mucus illustrated as a maximum intensity projection. **a** “Fresh” mucus showing early colonization by bacteria. **b** Mucus after 28 days with bacteria (red) and protists (green) binding the lectin at their cell surface (large spherical cells, presumably *T. pyriformis*). Colour allocation: lectin = green, nucleic acids = red, the yellow signals in **a** indicate co-localization of both fluorochromes

CLSM of fresh samples revealed that the mucus is initially excreted from the flatworm as a thin homogeneous layer about 15 µm thick, but that it quickly breaks down and becomes twisted (Fig. 3a, b). In living flatworms, only the sides and tip of the tail region were covered with mucus (Fig. 3c). In the time-series, the volume occupied by bacterial cells growing on the mucus secretions increased over time (from 0.14 ± 0.5 at deposition to 0.39 ± 0.24 µm^3^ µm^−2^ after 28 days). Aggregation of the formerly thin mucus layer caused it to form a thicker (from 0.27 ± 0.07 at deposition to 0.57 ± 0.13 µm^3^ µm^−2^ after 28 days) exopolysaccharidic matrix colonized by bacteria and in some cases with protists (compare Fig. 4a, b).

## Discussion

Our results show that nematode fitness is affected by flatworm mucus, but the effect is species dependent. Nematodes were able to sense mucus, and the mucus trails served as a durable structure and resource for other prokaryotic and eukaryotic organisms.

The inconsistency in the observed responses of the two nematode species to the flatworm’s mucus can be explained by the fact that, in nature, *C.* *elegans* is not exposed to the mucus secretions of aquatic flatworms, whereas *P.* *acuminatus* is a free-living species that naturally coexists with *P. tenuis*. *P.* *acuminatus* is a slower reproducer and has a much longer lifespan than *C.* *elegans* (generation time of 3.8 days vs. 26.8 days) [17, 18]. In culture, it tolerates bacterial densities that are one order of magnitude lower than those tolerated by *C.* *elegans*. This would explain why the slight changes in bacterial density induced by the mucus had greater consequences for the reproduction and growth of *P.* *acuminatus*. It also accounts for the mixed response of nematode fitness to mucus secretions, with no effect on *C.* *elegans* whereas *P.* *acuminatus* was positively affected. Nevertheless, the cafeteria experiment showed that both species were attracted by bacterial patches, regardless of the presence of mucus, which ruled out de facto potentially deterrent effects of mucus compounds on nematode feeding behavior. In previous studies, *Tetrahymena pyriformis*, a well-studied bacterivorous ciliate and the dominant protozoan in this study, occurred at similar densities as in the experiments described herein [19, 20]. It is also likely that *T. pyriformis* is a commensal or facultative parasite of flatworms (in agreement with the observations of Wright [21]), since introduction of the ciliate into our experimental system could only have occurred by attachment to the flatworms. The presence of *T. pyriformis* dampened the production of nematode juveniles, suggesting that either the protozoan was a superior competitor for bacterial food or it reduced nematode reproduction directly by feeding on nematode eggs. Bergtold et al. [22, 23] found evidence of intense interspecific competition between nematodes and ciliates and showed that nematodes were, at least temporarily, strongly affected.

The effects of mucus secretions on bacterial growth are unclear. While Cruickshank [24] failed to find an antibiotic effect of flatworm mucus, Calow [25] suggested that bacterial inhibition is essential for flatworm mucus to remain viscid and able to trap prey. Our observations rule out a potential negative effect of mucus on bacterial growth (Fig. 4). Although different types of mucus might be excreted by flatworms, the mucus tested here was mostly related to facilitating locomotion and food trapping. The production of a mucus with additional antibiotic properties may be energetically too costly. Instead, the thin layers of mucus excreted from the tail of *P. tenuis* were quickly colonized by bacteria and protozoans (and nematodes) and thereby formed an architectural basis for the emergence of a complex, patchy biofilm. Thus, the influence of mucus can persist for weeks after flatworm passage, in good agreement with the report of Calow [25], in which the viscid properties of the mucus from the freshwater triclad flatworm *Dendrocoelum lacteum* were shown to persist for over 16 days. The adhesive properties of mucus offers an advantage by allowing passive hunting. Little is known about the composition of Flatworm mucus except that it contains carbohydrates and that ca. 11% of its dry weight is made up of various proteins, including potent enzymes [26, 27]. While the proteinaceous nature of the mucus of *P. tenuis* could not be confirmed, the detection of glycoconjugates suggested that the mucus provides a relevant resource for bacterial growth and for the establishment of a food web. As such, it serves as a valuable reservoir of bacterivorous prey for “gardening” flatworms.

## Conclusions

In summary, our results indicate that ecological engineering, and specifically that of the mucus excreted by predatory flatworms, can modify prey fitness, although in this study the mechanism was primarily mediated by an effect on non-prey microbes. Nematodes and microorganisms were affected by the mucus, in terms of their fitness and interspecific interactions. The durability of the mucus suggested that the enhanced environment of the flatworms is capable of sustaining future generations of these organisms.

## Methods

### Culture procedures

*Polycelis tenuis* flatworms were collected from ponds using a weir trap consisting of a 50-mL polyethylene tube and a cut pipette tip (entrance diameter = 2 mm) submerged with a dead cricket as bait. After 24 h, the collected flatworms were transferred into glass jars (volume = 1700 mL, diameter = 12 cm) at a density of 15 flatworms per jar. Each jar contained a tile with an edge length of 5 cm and 1500 mL of filtered tap water (pH = 7.55, temperature = 20 ± 2 °C). The water was aerated, and half of the volume renewed weekly. The flatworms were fed a pea-sized piece of raw pork once a week, with the remains of the previous meal removed before each new feeding. The jars were passively illuminated under a 12:12-h light:dark regime (photon flow density of 0.1 μmol m^−2^ s^−1^). Under these conditions, the flatworms thrived, as evidenced by their reproduction between experiments. An average of one cocoon per individual was observed and hatched offspring were released.

*Caenorhabditis elegans* var. Bristol, strain N2 was maintained as stocks of dauer larvae on nematode growth medium (NGM) agar (500 mL deionized water, 17 g bacto-agar, 2.5 g bacto-peptone, and 3 g NaCl L^−1^; after autoclaving, the following were added: 1 mL of 1 M CaCl_2_,1 mL of 1 M MgSO_4_, 25 mL of 1 M KH_2_PO_4_, and 1 ml of 5 mg cholesterol/mL, prepared in ethanol) and were cultured and handled according to DIN ISO 10872 (International Organization for Standardization 2010). Synchronized adults were obtained by transferring synchronized first-stage (J1) juveniles to a new agar plate and used for experiments at the emergence of the first eggs.

*Plectus acuminatus* was cultured as described for *C.* *elegans* but the agar was replaced with water nematode growth gerlite (WNGG) medium (1.25 g gerlite, 0.167 MgSO_4_·7H_2_O, and 250 mL deionized water; after autoclaving, 250 µL of 5 mg cholesterol/mL, prepared in ethanol, was added). A preparation of *E. coli* (200 µL) was spread on the surface as a food source. After 2 weeks, the J3 individuals used for the experiment were manually selected after sieving the culture through a 35-µm mesh.

### Effects of mucus on nematode fitness: Nematode bioassay

The standard nematode bioassay originally described for *C. elegans*, Maupas 1900 (DIN ISO 10872 [28]) was carried out with a few modifications. Flatworms of the species *P. tenuis* were caught in a small pond in the campus area of the University of Bielefeld and kept in culture in the laboratory as described above. All experiments were performed using flatworms with a body length of 10 ± 2 mm. Before the experiments, the flatworms were starved in filtered tap water for 48 h to avoid excretion effects.

Three treatments were used to account for differences in the composition of mucus secreted on the different substrates and in the presence or absence of bacteria. In the first treatment (1st mucus + 2nd *E. coli*), one flatworm was transferred into a glass vial (12 mL, diameter = 24 mm) containing 1 mL of minimally mineralized, commercial Volvic water and gently removed after 0 (control without flatworm), 1.25, 5, 20, 80, 320, or 1280 min. The movements of the worm were filmed for the first 80 min to compare estimated vs. observed active periods (see Additional file 1). Seven replicates were used for each time point. After the removal of the flatworms, the water in the vial was replaced with 500 µL of Volvic and 500 µL of *E. coli* suspension (1000 FAU suspended in Volvic and 0.02% vol. of 5 mg cholesterol/mL, prepared in ethanol). The second treatment (1st *E. coli* + 2nd mucus) was similar, except that the flatworms were placed directly in 500 µL of Volvic and 500 µL of *E. coli* suspension. In the third treatment (1st *E. coli* and sediment + 2nd mucus), 500 µL of Volvic and 500 µL of a “concentrated” *E. coli* suspension (12,000 FAU suspended in Volvic and 0.02% vol. 5 mg cholesterol/mL prepared in ethanol) were mixed with 0.4 g of quartz sand (particle size: 0.6–1.2 mm) prior to mucus secretion.

After removal of the flatworms, the vials were stored overnight in the dark at 8 °C, after which ten J1 *C.* *elegans* juveniles (see Additional file 1 for culture details) were transferred to each test vial and to the control vials without mucus. After incubation for 96 h at 20 °C, the test was stopped by heat-killing the nematodes at 70 °C. When needed, nematodes were extracted from the sediment according to DIN ISO 10872 [28]. The nematodes were stained in dishes containing 0.5 mL of an aqueous solution of Rose Bengal (0.5 g L^−1^) and then counted and measured at 32× magnification under a dissecting microscope (Leica MZ 125).

Nearly half of the *P. tenuis* individuals were naturally colonized by protozoans (*Tetrahymena pyriformis*); hence a protozoan inoculum was equally distributed across vials incubated for > 1.25 min. This was considered as an additional protozoan treatment in the experiment. Protozoan populations occurred at a relatively constant density of 50 ± 7.5 × 10^3^ individuals mL^−1^.

For the bioassay with *P.* *acuminatus*, 20 Petri dishes (diameter = 4.5 cm) were filled with 2 mm of WNGG medium (because of the surface tension, 10 mL were inserted, and 5 mL carefully removed thereafter). After the medium had solidified, 500 µL of an *E. coli* suspension (1000 FAU suspended in Volvic and 0.02% vol. 5 mg cholesterol/mL, prepared in ethanol) was added. Roughly 30 min later, when the bacteria had either caved-in or attached to the surface, 5 mL of Volvic was added to the surface. In ten of the dishes, the flatworms were allowed to secrete mucus for 80 min. Since *P.* *acuminatus* is a slower reproducer than *C. elegans*, the inoculation and incubation protocols were as follows: 50 *P.* *acuminatus* juveniles (J3) were added to the dishes, which were then sealed with Parafilm and incubated in the dark for 2 weeks at 20 °C. The test was stopped by heat-killing the nematodes at 70 °C. The WNGG medium was dissolved using 0.1 M EDTA and the contents of the dish were sieved to obtain the nematodes, which were then stained and processed as described for *C.* *elegans*.

### Effects of mucus on nematode food choice: cafeteria experiment

“Cafeterias” were established using Petri dishes (diameter = 8 cm) filled with agar (1 L Volvic, 17 g bacto-agar) in which six equidistant wells were punched out using a centrifuge tube (diameter = 1.5 cm). The wells were then filled with 125 µL of agar with or without the following food sources: Volvic, mucus in Volvic, mucus in Volvic and replacement of the water after flatworm contact, mucus in Volvic and replacement of the water with an *E. coli* suspension (10^9^ cells mL^−1^) after flatworm contact, mucus in *E. coli* suspension and pure *E. coli* suspension (Additional file 1: Fig. S3). The arrangement of each food source was randomized. Before the wells were filled with 100 µL of each food source, the agar surface was wiped with a wet (deionized water) sterile tissue (Rotizell, Roth, Germany) to create a homogeneous film of water. The run was started by placing ten active adults of *C. elegans* or *P. acuminatus* in the middle of the cafeteria. The experiment was conducted at ambient laboratory conditions (23 °C, photon flux: 48.1 μmol m^−2^ s^−1^). After the dishes were Parafilm-sealed, the number of nematodes that had moved into the wells was determined every 30 min for 3 h and then every 60 min for the next 3 h using a dissection microscope (32× magnification).

### Structural analysis of the mucus trails

To investigate its structure and colonization by microorganisms over time, the mucus was examined using confocal laser scanning microscopy (CLSM). The flatworms were allowed to crawl for 80 min on polycarbonate slides (n = 25, 2 × 2 cm) covered by a drop of Volvic water. The flatworms were then removed, and the slides incubated under the same conditions used in the flatworm cultures (for details see Additional file 1), with either 15 mL of Volvic water, pond water (filtered through a 5-µm mesh), Volvic + 1 mL *E.* *coli* suspension (10^9^ cells mL^−1^), or Volvic + 1 mL *E.* *coli* suspension + ca. 1000 adult *C.* *elegans*. After 7, 14, 21 and 28 days of incubation, the slides were preserved using 6% formalin. Fresh mucus secretions were elicited immediately before microscopy using the same approach but without preservative. Living *P. tenuis* individuals were also observed by CLSM. For microscopy, the slides were directly placed in 5-cm diameter dishes, stained and observed under an upright confocal microscope controlled by the software LAS-AF ver. 2.7.3 (TCS SP5X AOBS Leica, Germany). For imaging, a 25 × NA 0.95 water-immersible objective lens was employed. Optical sections were usually collected at 0.5-µm step size.

For staining the glycoconjugates of the mucus, a panel of lectins was tested: AAL-A488, Ban-FITC, RCA-FITC (Vector Laboratory), GS-I-FITC, HPA-FITC, PSA-FITC, VVA-FITC (EY Labs) and WGA-FITC (Sigma). Images were recorded with the following settings: Excitation 490 nm, emission 505–580 nm (FITC and Alexa488 lectins), excitation 650 nm, emission 675–750 nm (Syto60). Additionally, the nucleic acid specific fluorochrome Syto60 was used as a counterstain bacteria. SyproOrange was tested to stain protein but was not consistently applied due to low protein concentration in mucus and outshining by the lectin.

For representation, z-stacks of CLSM images were projected as maximum intensity projections (MIPs). The area (pixels) occupied by nucleic acids, as stained by S60, and by the glycoconjugate matrix, as stained by RCA-FITC, was measured in each stack, and then further converted to biovolume by taking z-stack intervals into account.

### Statistical analyses

All statistical analyses were done using R version 3.3.3 [29]. The data were checked for normality using the Shapiro–Wilk test, and the homogeneity of variance using the Levene test. The Kruskal–Wallis test was used to analyze the data from the nematode bioassay using *P.* *acuminatus*. Significant results were then further analyzed using a pairwise Mann–Whitney-U-test with a Bonferroni-Holm correction.

The *lme4* package [30] was used for modeling and the *MuMIn* package [31] to obtain the R^2^ values for the models. The *Lattice* package [32] was used to check residuals. According to the Cooks’ distances, no conspicuous data were found or excluded. The P-value was obtained in likelihood ratio tests. Vial or Petri dish numbers were always used as the random effect to control for possible dependence due to repeated measures or order effects. Although most of the random effects had very little variance and were not significant according to the *RLRsim* package [33], they were included for a wider inference and to more faithfully represent the actual study design, as recommended by Littell et al. [34].

Linear mixed effect models were used in the nematode bioassay, with juveniles or nematode body length as the response variable. Time, treatment and protozoans were used as fixed effects for the length. Protozoans served as a random effect for the juveniles produced per capita, due to the small sample size. In the food-choice experiments, the number of nematodes per well was set as the response variable, with time and food source as fixed effects. The analysis was followed by a post hoc Tukey HSD test. All significance thresholds were set to α = 0.05.

## Additional file

**Additional file 1.** Additional data on methods, preliminary tests, and the statistic.

## Abbreviations

CLSM: confocal laser scanning microscopy
Fig.: figure
J1: first stage juveniles
MIPs: maximum intensity projections
WNGG: nematode growth gerlite
NGM: nematode growth medium
