# Supplementary material for: Flatworm mucus as the base of a food web
Source: BMC Ecol. 2019 Mar 29;19:15. doi: 10.1186/s12898-019-0231-2 (PMC6441204; doi:10.1186/s12898-019-0231-2)
Supplement: Supplementary file 1 — Additional file 1. Additional data on methods, preliminary tests, and the statistic. [file 12898_2019_231_MOESM1_ESM.pdf]

Additional File 1

Estimated vs. observed active periods in Polycelis tenuis

The analysis of the movement times showed that the exposure times significantly correlated with observed movement (Spearman's rank correlation rho, S = 6521, P = 0.002) and that there was no difference between treatments (T-Test, t = -0.49869, P = 0.62). But the real movement time was almost every time considerably below the exposure time (see Fig. A1). Actually, even some of the flatworms which were 80 minutes in the vials hardly moved at all. The video analysis was performed in Fiji [1].

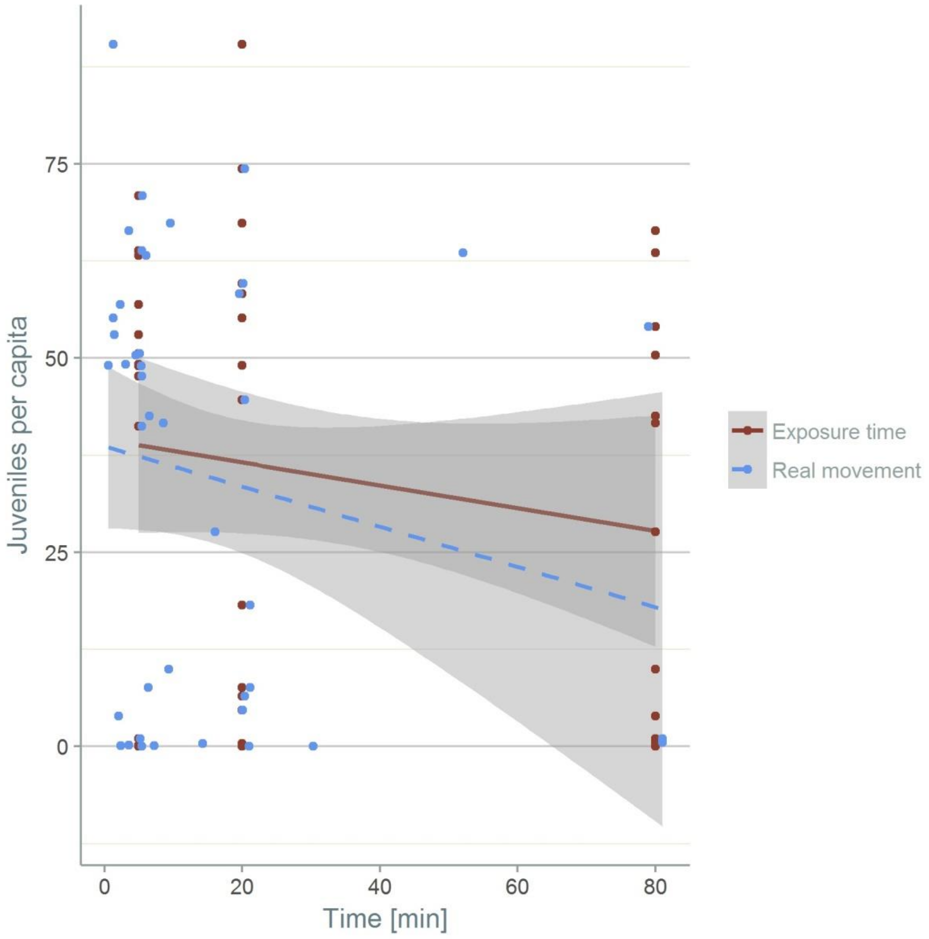

<u>Fig. S1:</u> Juveniles per capita of *C. elegans* in standard nematode bioassay with different exposure times and actual movement of *P. tenuis* with and without flatworm contact to *E. coli*

(N = 7 per time point and treatment). Besides the actual data, an LM fit is shown with the 95% confidence interval to show the correlation between movement and exposure time (Spearman's rank correlation rho, S = 6521, P = 0.002).

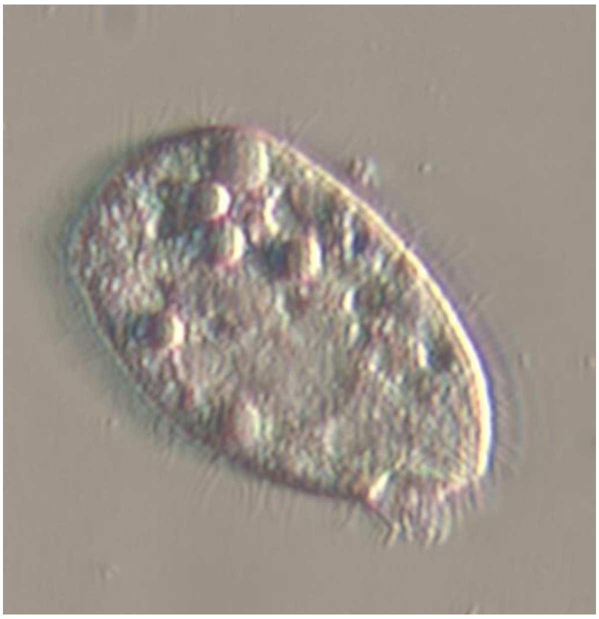

Fig. S2: *Tetrahymena pyriformis* from nematode bioassay.

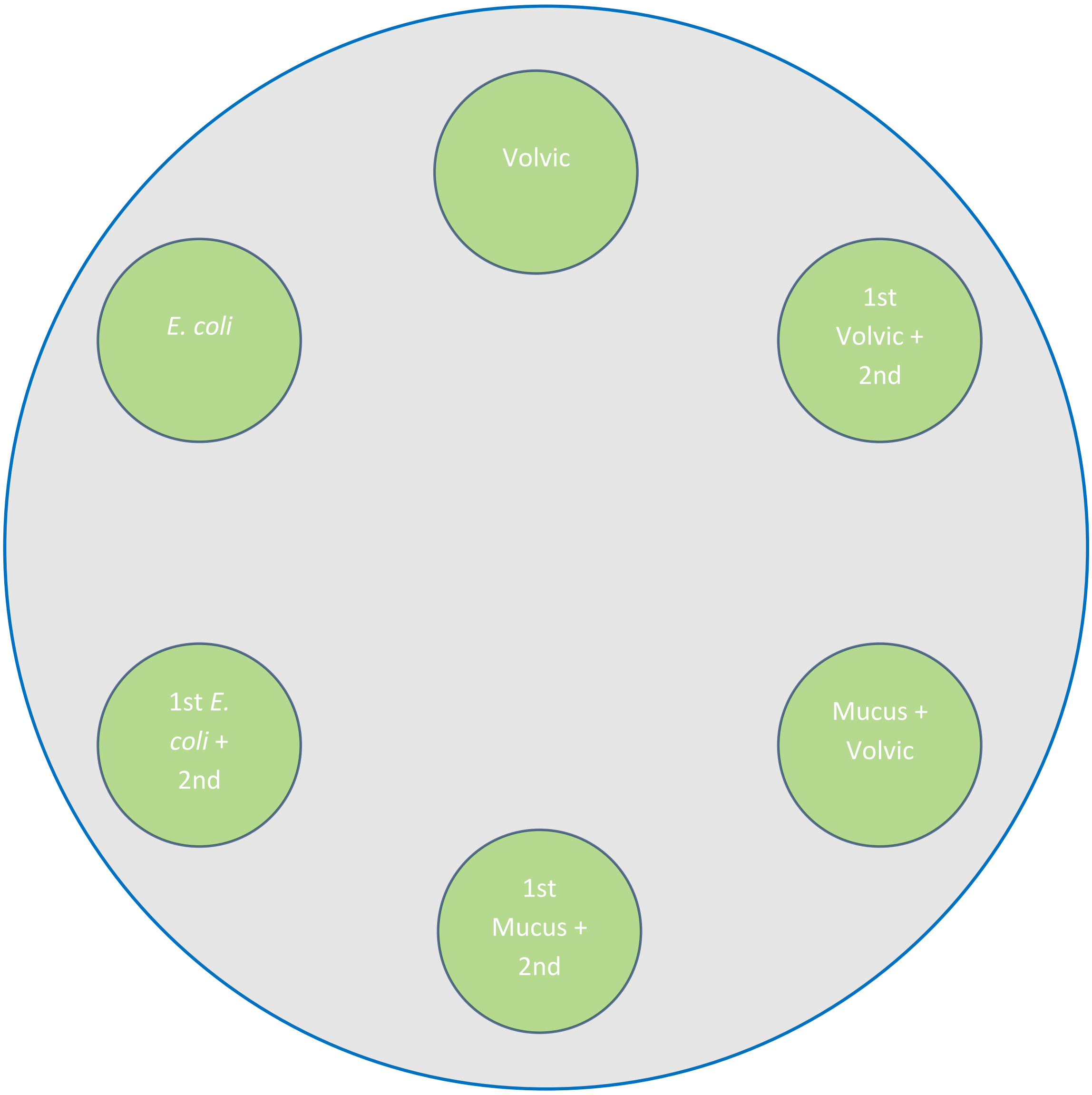

Fig. S3: Setup of the cafeteria experiment

Bacterial growth vs. antibiotic effects

The agar diffusion test resulted in no antibiotic effect, but huge contamination due to microorganisms introduced by flatworm mucus. The grown area is significantly larger than in the control (T-test, t = -2.4428, df = 21, p-value = 0.01).

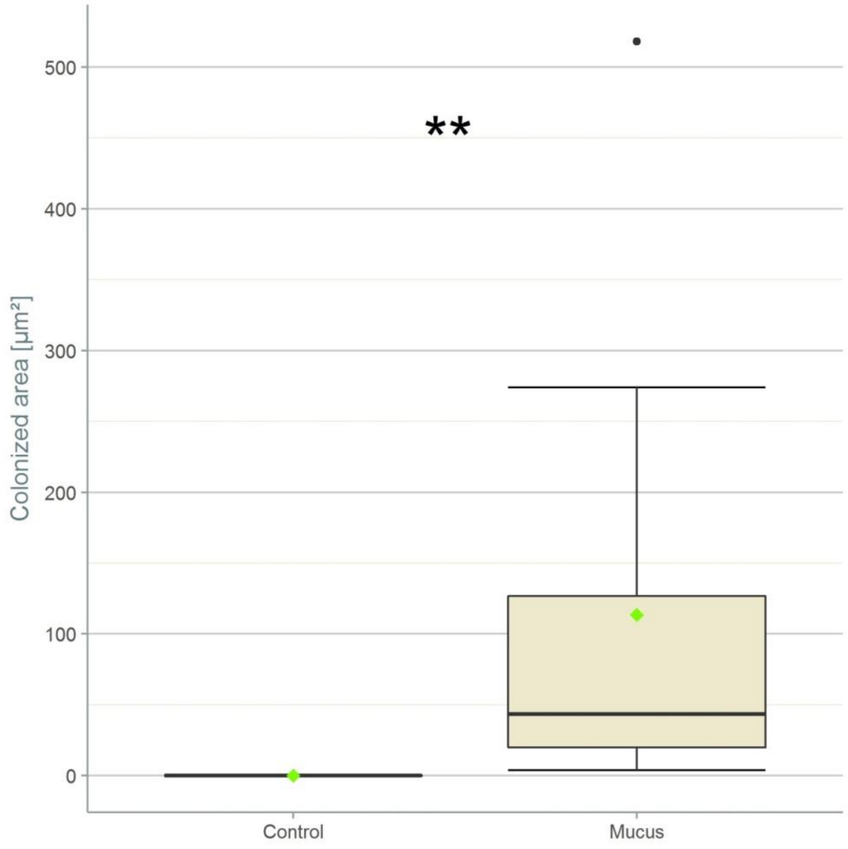

Fig. S4: Colonized area [µm] of various microorganisms introduced in a agar diffusion test by *P. tenuis* mucus on a *E. coli* culture on NGM agar. The data are presented in a Box-Whisker plot; the median is represented by the horizontal line, mean by the green rhombus, the boxes show the interquartile range and the whiskers illustrate either the 5 or the 95 % percentile.

Table. S1: Importance of different factors for juveniles per capita of *C. elegans* by LMM

Factor	X^2	P-value	Significance
**Time**	8.421	0.135	n.s.
**Treatment**	2.813	0.590	n.s.
**Time:Treatment**	2.812	0.245	n.s.

Table. S2: Importance of different factors for the body length of *C. elegans* by LMM

Factor	X^2	P-value	Significance
**Time**	1.916	0.166	n.s.
**Treatment**	1.967	0.374	n.s.
**Protists**	850.93	< 0.001	***
**Time:Treatment**	0.030	0.862	n.s.
**Time:Protists**	0.677	0.713	n.s.
**Protists:Treatment**	12.508	0.002	**
**Time:Protists:Treatment**	0.010	0.995	n.s.

Table. S3: Importance of different factors for number of individual *C. elegans* per well by LMM

Factor	X^2	P-value	Significance
**Time**	8.328	0.004	**
**Treatment**	275.02	< 0.001	***
**Time:Treatment**	5.420	0.367	n.s.

Table. S4: Importance of different factors for number of individual *P. acuminatus* per well by LMM

Factor	X^2	P-value	Significance
**Time**	11.68	< 0.001	***
**Treatment**	15.68	0.008	**
**Time:Treatment**	1.854	0.869	n.s.

<u>Table. S5:</u> Results of the post hoc Tukey HSD on the length of *C. elegans* in the second nematode bioassay with flatworms directly introduced to *E. coli* medium.

Time point	diff	lwr	upr	p adj	sign. Level
**5-Control**	-18,795	-115,829	78,239	0,994	
**20-Control**	-38,867	-135,123	57,389	0,855	
**80-Control**	93,110	-11,796	198,016	0,114	
**320-Control**	-95,328	-186,448	-4,208	0,034	*
**1280-Control**	-144,573	-241,607	-47,539	0,000	***
**20-5**	-20,072	-124,844	84,700	0,994	
**80-5**	111,905	-0,866	224,675	0,053	
**320-5**	-76,533	-176,607	23,540	0,243	
**1280-5**	-125,778	-231,265	-20,291	0,009	**
**80-20**	131,977	19,875	244,078	0,011	*
**320-20**	-56,461	-155,781	42,858	0,577	
**1280-20**	-105,706	-210,478	-0,934	0,047	*
**320-80**	-188,438	-296,162	-80,714	>0,001	***
**1280-80**	-237,683	-350,453	-124,912	>0,001	***
**1280-320**	-49,244	-149,318	50,829	0,718	

<u>Table. S6:</u> Results of the post hoc Tukey HSD on the cafeteria experiment with *C. elegans.*

Food source	diff	lwr	upr	p adj	sign. Level
E. coli* + Muc- *E. coli	1,478	0,849	2,107	>0,001	***
**Mucus - *E. coli***	-1,678	-2,307	-1,049	>0,001	***
**Muc + E. coli - *E. coli***	0,200	-0,429	0,829	0,944	
**Mucus WC- *E. coli***	-1,544	-2,173	-0,916	>0,001	***
**Volvic - *E. coli***	-1,722	-2,351	-1,093	>0,001	***
**Mucus - *E. coli* + Muc**	-3,156	-3,784	-2,527	>0,001	***
**Muc + *E. coli* - *E. coli* + Muc**	-1,278	-1,907	-0,649	>0,001	***
**Mucus WC- *E. coli* + Muc**	-3,022	-3,651	-2,393	>0,001	***
**Volvic - *E. coli* + Muc**	-3,200	-3,829	-2,571	>0,001	***
**Muc + E. coli - Mucus**	1,878	1,249	2,507	>0,001	***
**Mucus WC- Mucus**	0,133	-0,496	0,762	0,991	
**Volvic - Mucus**	-0,044	-0,673	0,584	1,000	
**Mucus WC-Muc + *E. coli***	-1,744	-2,373	-1,116	>0,001	***
**Volvic -Muc + *E. coli***	-1,922	-2,551	-1,293	>0,001	***
**Volvic-Mucus WC**	-0,178	-0,807	0,451	0,966	

Table. S7: Results of the post hoc Tukey HSD on the cafeteria experiment with *P. acuminatus.*

Food source	diff	lwr	upr	p adj	sign. Level
E. coli* + Muc- *E. coli	0,080	-0,203	0,363	0,966	
**Mucus - *E. coli***	-0,150	-0,433	0,133	0,653	
**Muc + E. coli - *E. coli***	0,200	-0,083	0,483	0,331	
**Mucus WC- *E. coli***	0,080	-0,203	0,363	0,966	
**Volvic - *E. coli***	-0,150	-0,433	0,133	0,653	
**Mucus - *E. coli* + Muc**	-0,230	-0,513	0,053	0,185	
**Muc + *E. coli* - *E. coli* + Muc**	0,120	-0,163	0,403	0,830	
**Mucus WC- *E. coli* + Muc**	0,000	-0,283	0,283	1,000	
**Volvic - *E. coli* + Muc**	-0,230	-0,513	0,053	0,185	
**Muc + E. coli - Mucus**	0,350	0,067	0,633	0,006	**
**Mucus WC- Mucus**	0,230	-0,053	0,513	0,185	
**Volvic - Mucus**	0,000	-0,283	0,283	1,000	
**Mucus WC-Muc + *E. coli***	-0,120	-0,403	0,163	0,830	
**Volvic -Muc + *E. coli***	-0,350	-0,633	-0,067	0,006	**
**Volvic-Mucus WC**	-0,230	-0,513	0,053	0,185	

Literature cited

1. Schindelin J, Arganda-Carreras I, Frise E, Kaynig V, Longair M, Pietzsch T, et al. Fiji: an open-source platform for biological-image analysis. Nat Methods [Internet]. 2012;9:676–82. Available from: http://www.ncbi.nlm.nih.gov/pubmed/22743772%0Ahttp://www.pubmedcentral.nih.gov/articlerender.fcgi?artid=PMC3855844
